# Home Literacy Environment and Early Literacy Development Across Languages Varying in Orthographic Consistency

**DOI:** 10.3389/fpsyg.2020.01923

**Published:** 2020-07-31

**Authors:** Tomohiro Inoue, George Manolitsis, Peter F. de Jong, Karin Landerl, Rauno Parrila, George K. Georgiou

**Affiliations:** ^1^Department of Psychology, Chinese University of Hong Kong, Shatin, Hong Kong; ^2^Department of Preschool Education, University of Crete, Rethymnon, Greece; ^3^Faculty of Social and Behavioural Sciences, University of Amsterdam, Amsterdam, Netherlands; ^4^Institute of Psychology, University of Graz, Graz, Austria; ^5^Department of Cognitive Science, Macquarie University, Sydney, NSW, Australia; ^6^Department of Educational Studies, Macquarie University, Sydney, NSW, Australia; ^7^Department of Educational Psychology, University of Alberta, Edmonton, AB, Canada

**Keywords:** emergent literacy skills, home literacy environment, longitudinal, orthographic transparency, reading fluency, spelling

## Abstract

We examined the relation between home literacy environment (HLE) and early literacy development in a sample of children learning four alphabetic orthographies varying in orthographic consistency (English, Dutch, German, and Greek). Seven hundred and fourteen children were followed from Grade 1 to Grade 2 and tested on emergent literacy skills (vocabulary, letter knowledge, and phonological awareness) at the beginning of Grade 1 and on word reading fluency and spelling at the end of Grade 1, the beginning of Grade 2, and the end of Grade 2. Their parents responded to a questionnaire assessing HLE [parent teaching (PT), shared book reading (SBR), access to literacy resources (ALR)] at the beginning of Grade 1. Results showed first that PT was associated with letter knowledge or phonological awareness in Dutch and Greek, while ALR was associated with emergent literacy skills in all languages. SBR did not predict any cognitive or early literacy skills in any language. Second, PT and ALR had indirect effects on literacy outcomes via different emergent literacy skills in all languages. These findings suggest that not all HLE components are equally important for emergent literacy skills, reading fluency, and spelling. No specific trend in the role of orthographic consistency in the aforementioned relations emerged, which suggests that other factors may account for the observed differences across languages when children start receiving formal reading instruction in Grade 1.

## Introduction

Bronfenbrenner’s (1979, 1995) ecological systems theory of human development emphasizes that in order to understand children’s development we need to consider multiple levels of analyses that encompass the child and both proximal, microsystem (e.g., family and school) and distal, macrosystem (e.g., language and culture) environmental factors. In light of this, it is not surprising that previous studies have shown a significant effect of home literacy environment (HLE) on children’s language and literacy development across a variety of languages and cultural contexts (e.g., de Jong and Leseman, 2001; Silinskas et al., 2012, 2020; Niklas and Schneider, 2013; Inoue et al., 2018; Liu et al., 2018; Zuilkowski et al., 2019). However, despite a growing body of literature on the relations between HLE and children’s literacy skills, most existing studies on HLE have been conducted in a single language and cultural context, thereby focusing primarily on microsystem factors only. Indeed, only a few cross-linguistic studies have been conducted and they have been pairwise comparisons between English and another language (e.g., Bruck et al., 1997; Manolitsis et al., 2009; LeFevre et al., 2010; Niklas et al., 2015). No studies have compared longitudinally the relations between HLE and children’s literacy skills across multiple cultures and orthographies varying in consistency (Silinskas et al., 2020). Given the reported differences in the frequency of distinct home literacy activities across languages, it remains unclear whether the relations between these HLE components and literacy skills also vary across languages. Thus, in the current study, we examined the role of HLE in early literacy development in a sample of children learning four European alphabetic orthographies varying in orthographic consistency (English, Dutch, German, and Greek).

### The Home Literacy Model

To date, most studies on HLE have been guided by the Home Literacy Model (Sénéchal and LeFevre, 2002; Sénéchal, 2006; Sénéchal et al., 2017), according to which parent–child interactions during home literacy activities are grouped into two categories: code-related (formal) and meaning-related (informal) activities. Code-related activities engage children directly with print through activities such as teaching of reading and spelling. In contrast, meaning-related activities are those for which the meaning carried by the print is the main focus of the activities, such as parents’ shared book reading with their children (Sénéchal, 2006). Code-related activities have usually been assessed in terms of frequency of parents’ teaching of letters/words, while meaning-related activities have usually been assessed in terms of frequency of shared book reading and access to literacy resources (including the number of children’s books at home; see e.g., Sénéchal, 2006; Sénéchal and LeFevre, 2014). Several studies have shown that (a) code-related activities are associated with later reading through letter knowledge and (b) meaning-related activities are associated with later reading through oral language skills including vocabulary (for a review, see Sénéchal et al., 2017).

Previous longitudinal studies have consistently supported these predictions across a wide range of linguistic and cultural contexts (e.g., Lehrl et al., 2013; Manolitsis et al., 2013; Sénéchal and LeFevre, 2014; Hamilton et al., 2016; Niklas and Schneider, 2017a). For example, in a longitudinal study with a sample of English-speaking Canadian children, Sénéchal and LeFevre (2014) found that shared book reading during the kindergarten year predicted growth in receptive vocabulary from kindergarten to Grade 1, whereas the frequency of parent teaching of reading predicted growth in early literacy from kindergarten to Grade 1 and growth in word reading during Grade 1. Similarly, Silinskas et al. (2010a) found that mothers’ teaching of reading predicted the development of reading skills among Finnish kindergarten children.

### Cross-Linguistic Studies on HLE

Existing cross-linguistic studies have also provided evidence in support of the important role of HLE in literacy acquisition across languages (Bruck et al., 1997; Manolitsis et al., 2009; Niklas et al., 2015). For example, in a cross-linguistic study with a sample of English- and Greek-speaking children, Manolitsis et al. (2009) found that parents’ teaching of letter names and sounds at home (called direct teaching) was associated with letter knowledge in both languages. Niklas et al. (2015) in turn found the associations between home-based literacy activities and children’s verbal and cognitive abilities in English-speaking Australian and German children. Moreover, cross-cultural studies based on international survey data [e.g., Progress in International Reading Literacy Study (PIRLS); Program for International Student Assessment (PISA)] have consistently shown robust relationships between the amount of reading materials at home and children’s early literacy skills across sociocultural contexts (Chiu et al., 2012; Arya et al., 2014; Araújo and Costa, 2015; Lenkeit et al., 2018; Zuilkowski et al., 2019).

Despite the consistent evidence of positive associations between HLE and children’s literacy development (e.g., Hood et al., 2008; Kirby and Hogan, 2008; Silinskas et al., 2010b; Manolitsis et al., 2011; Dulay et al., 2018), the existing studies have some important limitations. First, most previous cross-linguistic studies were pairwise comparisons between English and one other language (Bruck et al., 1997; Manolitsis et al., 2009; Niklas et al., 2015). Indeed, we are not aware of any study directly comparing these relationships between more than two languages varying in orthographic consistency. Additionally, the few cross-linguistic studies that included more than two languages have assessed relatively limited aspects of HLE (e.g., number of books at home) and have covered only one grade level (e.g., Grade 4; see Chiu and McBride-Chang, 2006, 2010). Second, because many previous studies have assessed meaning-related HLE in terms of both frequency of shared book reading and access to literacy resources, it remains unclear whether it is the former or the latter that is driving the relation between meaning-related HLE and children’s vocabulary knowledge. In fact, recent studies have shown that access to literacy resources can be a separable construct from shared book reading (e.g., Dulay et al., 2018; Esmaeeli et al., 2019; Zhang et al., 2019) and plays a unique and important role in children’s literacy development over and above parent teaching and shared reading (e.g., van Bergen et al., 2017; Vasilyeva et al., 2018; Zuilkowski et al., 2019). Finally, the main focus of previous research on HLE has been on its relationship with reading (e.g., Chiu and McBride-Chang, 2006; Arya et al., 2014; Araújo and Costa, 2015), and there is a dearth of research examining the relationship between HLE and spelling development across languages. This is important as many parents engage not only in reading activities with their children but also in writing activities. For example, Aram and Levin (2001, 2004) have shown that maternal writing mediation in kindergarten predicts children’s literacy outcomes in school beyond the early literacy measures assessed in kindergarten.

### The Present Study

In this study, we examined the developmental relations between HLE and literacy skills in a 2-year longitudinal study with children learning four European alphabetic orthographies varying in orthographic consistency: English, Dutch, German, and Greek. These languages were selected to vary widely in their orthographic consistency, namely, English being the most inconsistent, Greek being the most consistent, and Dutch, and German lying in between English and Greek in the orthographic consistency continuum (Seymour et al., 2003; Borgwaldt et al., 2004). Guided by the Home Literacy Model and the previous findings from within- and cross-language studies reviewed above, we expected that (a) parents’ teaching of reading and spelling (the code-related activities) would predict letter knowledge and phonological awareness in all languages (Lehrl et al., 2013; Manolitsis et al., 2013; Hamilton et al., 2016; Silinskas et al., 2020), and their association would be stronger in English than in the other languages because children learning to read in English might need more elaborate teaching as its inconsistent grapheme-phoneme associations cannot be acquired through simple paired associate learning as in consistent orthographies (Manolitsis et al., 2009); (b) shared book reading (the meaning-related activities) would predict vocabulary in all languages (Manolitsis et al., 2013; Sénéchal and LeFevre, 2014; Inoue et al., 2018; Krijnen et al., 2020; Lehrl et al., 2020), but their association would be limited when access to literacy resources is taken into account separately (van Bergen et al., 2017; Zhang et al., 2019); (c) access to literacy resources would be uniquely associated with literacy skills over and above the effects of parent teaching and shared book reading and its effect would be similar across languages (Chiu and McBride-Chang, 2006; Araújo and Costa, 2015), and (d) all of the HLE aspects would have mediated effects on later reading and spelling via emergent literacy skills in all languages (Hamilton et al., 2016; Inoue et al., 2018; Lehrl et al., 2020).

## Materials and Methods

### Participants

Our sample consisted of 714 children followed from the beginning of Grade 1 until the end of Grade 2. One hundred and seventy-two children (82 girls [47.7%]; *M*_*age*_ = 75.87 months at the first measurement point) were native speakers of English and were recruited from six public elementary schools in Edmonton, Canada; 120 children (63 girls [52.5%]; *M*_*age*_ = 78.52 months at the first measurement point) were native speakers of Dutch and were recruited from five public elementary schools in Amsterdam, the Netherlands; 184 children (85 girls [46.2%]; *M*_*age*_ = 79.12 months at the first measurement point) were native speakers of German and were recruited from five public elementary schools in Graz, Austria; and 238 children (120 girls [50.4%]; *M*_*age*_ = 76.10 months at the first measurement point) were native speakers of Greek and were recruited from six public elementary schools in Heraklion, Greece. Our participants were recruited on a voluntary basis (letters of information were sent to the parents of all children attending Grade 1 in the participating schools) and were tested four times: at the beginning and end of Grade 1, and at the beginning and end of Grade 2. By the end of Grade 2, our sample consisted of 157 English-speaking (9% attrition), 107 Dutch-speaking (11% attrition), 167 German-speaking (9% attrition), and 219 Greek-speaking (8% attrition) children. In all countries, children start school at 6 years of age. The children in each site came mostly from families of middle socioeconomic background (based on the location of the schools and parents’ education), and none were experiencing any intellectual, emotional, or sensory difficulties. Parental and school consent was obtained prior to testing.

### Measures

#### Parent Teaching

Two 5-point Likert scale questions were used to assess parent teaching. The first asked “When your child was in Kindergarten, how often did you (or someone else at home) teach him or her to read words?” and parents responded on a scale ranging from *Never* (0 points) to *Daily* (4 points). The other question was worded similarly but replaced “to read words” with “to spell words.’’

#### Shared Book Reading

Two 5-point Likert scale questions were used to assess shared book reading. The first asked “When your child was attending Kindergarten, how many hours did you (or someone else) read to your child on a typical weeknight (Monday to Friday)?” and parents responded on a scale ranging from *Less than 5 min a day* (0 points) to *2 h or more* (4 points). The other question was worded similarly but replaced “on a typical weeknight (Monday to Friday)” with “on the weekend (Saturday and Sunday).”

#### Access to Literacy Resources (ALR)

To assess ALR, we first asked parents to report how many children’s books they had at home by using a 5-point scale (0 = none, 1 = 1–20, 2 = 21–60, 3 = 61–150, and 4 = more than 150 books). Second, we asked parents to report how many adult’s books they had at home by using a 5-point scale (0 = less than 100, 1 = 100–299, 2 = 300–499, 3 = 500–1000, and 4 = more than 1000 books).

#### Letter Knowledge

Letter-Sound Knowledge task was administered in each language. Although we also assessed Letter-Name Knowledge, it was at the ceiling in English and for this reason, we only used Letter-Sound Knowledge in this study. Children were shown each of the uppercase letters on an A4 paper and asked to say what sound each made; short vowel sounds were accepted for vowel letters, and consonant sounds with the following vowel for consonants. The score was the number of correct letter-sounds produced. The maximum score was 26 in English, 24 in Dutch, 22 in German, and 24 in Greek. Reliability of this measure has been reported to be higher than 0.90 in each language.

#### Phonological Awareness (PA)

To assess PA, we used Phoneme Elision in each language. The task included four practice items and 24 experimental items designed so as to match items phonologically across languages (see Landerl et al., 2019, for more information). Children were presented with one item at a time and then asked to repeat it with a specified phonological unit deleted. The score was the total number correct. Raykov’s (2001) omega coefficients for each orthography ranged from 0.84 to 0.91.

#### Vocabulary

Expressive vocabulary from Wechsler Intelligence Scales for Children (WISC; Wechsler, 2003) was used to assess vocabulary. Children were asked to define words of increasing difficulty and their answer in each item was scored with 0 (incorrect), 1 (partly correct), or 2 (fully correct). A participant’s score was the sum of scores aggregated across all responded items.

#### Reading Fluency

To assess reading ability, we administered a word reading fluency task. We adapted existing reading fluency tasks in each language (English: Torgeson et al., 1999; Dutch: van den Bos et al., 1994; Brus and Voeten, 1995; German: Moll and Landerl, 2010; Greek: Georgiou et al., 2012) by arranging their items in four columns on a page. Children were asked to read as many words as possible within a 60-s time limit. A practice trial with eight words preceded timed testing to allow children to familiarize themselves with the task demands. A participant’s score was the total number of syllables in the correctly read words within the specified time limit. This scoring procedure was necessary because of differences in the length of the words included in each task across languages. Test–retest reliability has been reported to be higher than 0.85 for elementary school children (Brus and Voeten, 1995; Torgeson et al., 1999; Moll and Landerl, 2010; Georgiou et al., 2012).

#### Spelling

To assess spelling ability, we adopted an existing spelling to dictation task in each language (English: Wechsler, 2001; Dutch: Geelhoed and Reitsma, 1999; German: Moll and Landerl, 2010; Greek: Mouzaki et al., 2007). The tester first said a target word followed by a sentence in which the target word was embedded, and then repeated the target word. Children were then asked to write the target word in the space provided. The items in each language were ordered in terms of increasing difficulty and a discontinuation rule of six consecutive errors was applied. A participant’s score was the total number of correct responses. Internal consistency has been reported to be higher than 0.90 for elementary school children (Geelhoed and Reitsma, 1999; Wechsler, 2001; Mouzaki et al., 2007; Moll and Landerl, 2010).

### Procedure

Letter knowledge, PA, and vocabulary were assessed at the beginning of Grade 1 (Time 1), and word reading fluency and spelling were assessed at the end of Grade 1 (Time 2), the beginning of Grade 2 (Time 2), and the end of Grade 2 (Time 3). All testing took place in quiet rooms in the children’s school during school hours by trained research assistants. The tests were administered in one session lasting about 25 min. Administration and scoring were standardized across all children and languages.

### Statistical Analysis

**FIGURE 1:**
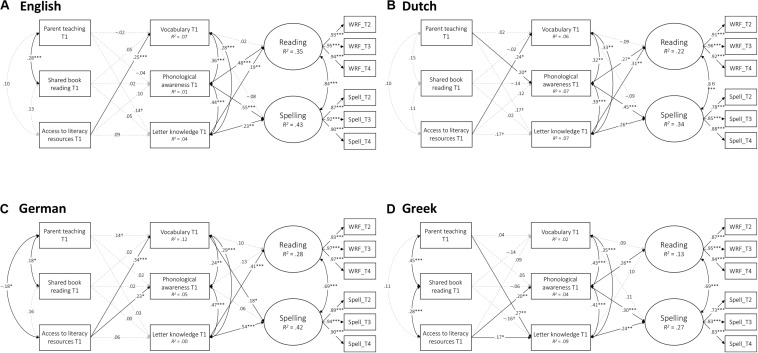
The models for the associations between HLE, emergent literacy skills, and later literacy outcomes in each orthography (standard solution): **(A)** English; **(B)** Dutch; **(C)** German; and **(D)** Greek. WRF, word reading fluency; T1, Time 1; T2, Time 3; T1, Time 3; T4, Time 4. ^†^*p* < 0.10, ^∗^*p* < 0.05, ^∗^*^∗^p* < 0.01, ^∗∗∗^*p* < 0.001.

First, to test the measurement equivalence of the latent HLE constructs across languages, we evaluated a model of metric invariance (Meredith, 1993), in which factor loadings were set to be equal across languages (Steenkamp and Baumgartner, 1998; Chen, 2007), using Mplus 8 (Muthén and Muthén, 1988–2017). To identify the model, the variance of each latent factor was fixed to 1 and the mean of each factor was fixed to 0. Second, to examine the relationships between HLE, children’s emergent literacy skills at the beginning of Grade 1 (Time 1), and literacy outcomes at the end of Grade 1 to the end of Grade 2 (Times 2–4), we constructed a longitudinal model (see Figure 1). Additionally, to test whether the associations between HLE and emergent literacy skills differ between languages, we performed multigroup analyses. Finally, to examine the indirect effect of HLE on later literacy outcomes, we conducted mediation analyses (MacKinnon et al., 2007; Hayes, 2013) using a bias-corrected bootstrapping technique with 2,000 resamples (Preacher and Hayes, 2008; Hayes and Scharkow, 2013).

All analyses handled missing data by the full information maximum likelihood estimator implemented in Mplus (Muthén and Muthén, 1988–2017), which has been found to result in trustworthy, unbiased estimates for missing values (Graham, 2009) and represents an adequate means of managing missing data in longitudinal study design (Jeličić et al., 2009). Model fits were examined using chi-square values and four fit indices: the comparative fit index (CFI), the Tucker-Lewis index (TLI), the root-mean-square error of approximation (RMSEA), and the standardized root-mean-square residual (SRMR). A non-significant chi-square value, CFI and TLI values above 0.95, RMSEA values below or at 0.06, and SRMR values below 0.08 indicate good model fit (Kline, 2015).

## Results

### Descriptive Statistics and Measurement Invariance

The descriptive statistics for parent measures are shown in Table 1. We first evaluated a model of metric invariance, in which factor loadings were set to be equal across languages. The results of CFA are shown in Table 2. The model showed an excellent fit, χ^2^ = 38.85, *df* = 33, *p* = 0.22, CFI = 0.99, TLI = 0.99, RMSEA = 0.04, 90% CI = 0.00 to 0.07, SRMR = 0.04, and the factor loadings were all substantial (all *p*s < 0.001; see Table 2). Additionally, there was no significant difference in the model fit between the measurement model and the model with free factor loadings for all paths (Δχ^2^ = 10.55, *df* = 9, *p* = 0.31). These results indicate that our HLE questionnaire showed measurement equivalence across the four languages. The results of one-way ANOVAs with language as a factor showed that parent teaching was more frequent in English than in all other orthographies (Hedges’ *g*s ranged from 0.84 to 1.04). Shared book reading was less frequent in Dutch (Hedges’ *g*s ranged from 0.62 to 0.89), while ALR was greater in German (Hedges’ *g*s ranged from 0.44 to 0.79) than in all other orthographies. The descriptive statistics for child measures are shown in Table 3 and the correlation matrices between all the variables for each orthography are shown in Table 4.

**TABLE 1:** Descriptive statistics for parent measures within each orthography.

	English			Dutch			German			Greek		
	*N*	*M*	*SD*	*N*	*M*	*SD*	*N*	*M*	*SD*	*N*	*M*	*SD*
Teach to read words^1^	172	3.06	0.94	89	1.94	1.06	128	1.84	1.38	174	1.91	1.23
Teach to print letters/words^1^	172	2.75	0.85	90	2.18	1.01	130	1.96	1.11	176	2.35	1.04
Read to child (weeknight)^2^	172	1.45	0.76	90	1.17	0.48	132	1.53	0.81	176	1.66	0.97
Read to child (weekend)^2^	172	1.56	0.81	90	1.08	0.52	132	1.92	0.92	176	1.67	0.94
Number of children’s books^3^	172	3.09	0.88	89	2.43	0.86	132	2.83	0.82	173	2.31	0.84
Number of adults’ books^4^	172	0.95	1.02	89	0.91	1.13	132	1.97	1.32	174	1.18	1.12

*^1^0 = Never, 4 = Daily; ^2^0 = Less than 5 min a day, 4 = 2 h or more; ^3^0 = none, 1 = 1–20, 2 = 21–60, 3 = 61–150, and 4 = more than 150 books; ^4^0 = less than 100, 1 = 100–299, 2 = 300–499, 3 = 500–1000, and 4 = more than 1,000 books.*

**TABLE 2:** The standardized factor loadings for the measurement model of the HLE questionnaire in each orthography.

	English			Dutch			German			Greek		
	PT	SBR	ALR	PT	SBR	ALR	PT	SBR	ALR	PT	SBR	ALR
Teach to read words	0.717			0.845			0.855			0.816		
Teach to print letters/words	0.707			0.831			0.844			0.793		
Read to child (weeknight)		0.642			0.658			0.678			0.634	
Read to child (weekend)		0.916			0.957			0.997			0.868	
Number of children’s books			0.650			0.756			0.870			0.837
Number of adults’ books			0.485			0.573			0.657			0.638
Internal consistency^a^	0.673	0.742	0.473	0.824	0.764	0.605	0.840	0.790	0.727	0.786	0.728	0.700

*PT, parent teaching; SBR, shared book reading; ALR, access to literacy resources. ^a^Spearman-Brown coefficient.*

**TABLE 3:** Descriptive statistics for child measures at beginning of grade 1, beginning of grade 2, and end of grade 2 within each orthography.

	English			Dutch			German			Greek		
	*N*	*M*	*SD*	*N*	*M*	*SD*	*N*	*M*	*SD*	*N*	*M*	*SD*
**Emergent literacy skills**												
Vocabulary T1	172	18.18	6.21	114	16.89	6.24	177	21.08	5.46	234	10.25	3.29
Elision T1	172	10.45	4.59	114	10.64	5.38	183	6.10	5.33	233	4.51	5.44
Letter knowledge T1	170	23.70	3.14	114	19.98	3.43	184	14.41	6.41	233	15.03	7.75
**Reading**												
WRF T2	170	56.16	29.37	113	39.44	28.99	175	45.72	26.66	229	58.89	26.36
WRF T3	161	72.11	33.20	108	64.12	35.08	170	65.26	33.38	224	77.83	35.14
WRF T4	157	91.52	30.22	107	85.09	34.04	167	85.45	37.77	219	104.15	39.68
**Spelling**												
Spelling T2	170	19.03	3.28	113	13.32	5.98	175	9.55	5.11	231	13.61	5.15
Spelling T3	159	20.52	3.81	108	17.90	6.76	170	12.61	5.09	224	28.84	5.22
Spelling T4	157	23.73	5.01	106	27.92	6.75	167	17.24	5.10	219	35.01	7.14

*WRF, word reading fluency; T1, Time 1; T2, Time 3; T3, Time 3; T4, Time 4.*

**TABLE 4:** Correlations among the observed variables for each orthography.

	1	2	3	4	5	6	7	8	9	10	11
**English (*N* = 172)**											
(1) PT_T1											
(2) SBR_T1	0.28**										
(3) ALR_T1	0.10	0.12									
(4) Voc_T1	0.02	0.07	0.25**								
(5) Elision_T1	−0.02	0.02	0.09	0.37**							
(6) LK_T1	0.18*	0.10	0.11	0.29**	0.44**						
(7) WRF_T2	0.04	0.07	0.05	0.25**	0.60**	0.43**					
(8) WRF_T3	0.09	0.03	0.09	0.21*	0.56**	0.41**	0.86**				
(9) WRF_T4	0.11	0.05	0.12	0.31**	0.58**	0.37**	0.87**	0.90**			
(10) Spelling_T2	−0.01	0.07	0.05	0.18*	0.54**	0.46**	0.79**	0.72**	0.66**		
(11) Spelling_T3	0.00	0.08	0.08	0.17*	0.58**	0.42**	0.80**	0.78**	0.74**	0.83**	
(12) Spelling_T4	−0.02	0.06	0.03	0.17*	0.59**	0.34**	0.78**	0.79**	0.75**	0.76**	0.82**
**Dutch (*N* = 120)**											
(1) PT_T1											
(2) SBR_T1	0.15										
(3) ALR_T1	0.09	0.11									
(4) Voc_T1	0.04	0.01	0.24*								
(5) Elision_T1	0.20^†^	−0.10	0.12	0.32**							
(6) LK_T1	0.19^†^	0.06	0.18	0.34**	0.39**						
(7) WRF_T2	0.14	−0.07	0.30**	0.13	0.38**	0.38**					
(8) WRF_T3	0.17	0.01	0.23*	0.14	0.31**	0.31**	0.85**				
(9) WRF_T4	0.20^†^	−0.06	0.23*	0.06	0.25*	0.30**	0.80**	0.86**			
(10) Spelling_T2	0.11	0.00	0.09	0.29**	0.52**	0.36**	0.65**	0.61**	0.53**		
(11) Spelling_T3	0.18	−0.06	0.13	0.08	0.46**	0.24*	0.61**	0.60**	0.53**	0.71**	
(12) Spelling_T4	0.19	−0.04	0.11	0.09	0.39**	0.34**	0.71**	0.72**	0.76**	0.64**	0.69**
**German (*N* = 184)**											
(1) PT_T1											
(2) SBR_T1	0.17										
(3) ALR_T1	−0.20*	0.16									
(4) Voc_T1	0.10	0.12	0.32**								
(5) Elision_T1	−0.01	0.03	0.21*	0.31**							
(6) LK_T1	0.01	−0.03	0.03	0.26**	0.45**						
(7) WRF_T2	−0.12	0.16	0.29**	0.21*	0.42**	0.40**					
(8) WRF_T3	−0.09	0.14	0.27**	0.22*	0.37**	0.38**	0.84**				
(9) WRF_T4	−0.07	0.21*	0.22*	0.22*	0.34**	0.37**	0.84**	0.95**			
(10) Spelling_T2	−0.11	0.08	0.31**	0.26**	0.43**	0.49**	0.67**	0.67**	0.66**		
(11) Spelling_T3	−0.03	0.04	0.28**	0.32**	0.42**	0.52**	0.65**	0.69**	0.70**	0.84**	
(12) Spelling_T4	−0.05	0.17	0.25*	0.26**	0.34**	0.44**	0.62**	0.67**	0.69**	0.77**	0.83**
**Greek (*N* = 238)**											
(1) PT_T1											
(2) SBP_T1	0.45**										
(3) ALR_T1	0.11	0.27**									
(4) Voc_T1	0.00	−0.08	0.07								
(5) Elision_T1	0.06	0.03	0.20*	0.40**							
(6) LK_T1	0.23**	0.01	0.15^†^	0.30**	0.44**						
(7) WRF_T2	0.07	−0.05	0.19*	0.26**	0.43**	0.33**					
(8) WRF_T3	0.07	−0.01	0.21*	0.29**	0.37**	0.26**	0.84**				
(9) WRF_T4	0.06	−0.03	0.12	0.24**	0.29**	0.22**	0.83**	0.91**			
(10) Spelling_T2	0.12	0.04	0.14^†^	0.22**	0.29**	0.25**	0.49**	0.51**	0.47**		
(11) Spelling_T3	0.11	−0.06	0.16^†^	0.31**	0.40**	0.31**	0.59**	0.62**	0.58**	0.55**	
(12) Spelling_T4	0.07	−0.02	0.18*	0.33**	0.44**	0.37**	0.62**	0.68**	0.67**	0.52**	0.69**

PT, parent teaching; SBR, shared book reading; ALR, access to literacy resources; Voc, vocabulary; LK, letter knowledge; WRF, word reading fluency. T1, Time 1; T2, Time 2; T3, Time 3; T4, Time 4. ^†^p < 0.10, *p < 0.05, **p < 0.01.

### Relations Between Home Literacy Environment, Children’s Emergent Literacy Skills, and Later Literacy Outcomes

Next, a longitudinal structural model was constructed (Figure 1). The model fit the data very well, χ^2^ = 269.38, *df* = 178, *p* < 0.001, CFI = 0.98, TLI = 0.96, RMSEA = 0.06, 90% CI = 0.05 to 0.08, SRMR = 0.05. Neither parent teaching nor shared book reading was significantly associated with emergent literacy skills in English or German, but parent teaching was in Dutch and Greek. ALR, on the other hand, was significantly associated with emergent literacy skills in all languages. More specifically, parent teaching was uniquely associated with children’s phonological awareness in Dutch (β = 0.20, *p* < 0.05) and letter knowledge in Greek (β = 0.27, *p* < 0.01). ALR was uniquely associated with vocabulary in English (β = 0.25, *p* < 0.001), Dutch (β = 0.24, *p* < 0.05), and German (β = 0.34, *p* < 0.001). ALR was also associated with PA in German (β = 0.23, *p* < 0.05) and both PA and letter knowledge in Greek (βs were 0.20, *p* < 0.01, and 0.17, *p* < 0.05, for PA and letter knowledge, respectively). In contrast, shared book reading did not have a unique association with any outcome measure.

The results of multigroup analyses showed that the fit of the multigroup model deteriorated significantly when the association between parent teaching and letter knowledge was constrained to be equal between German and Greek (Δχ^2^ = 4.30, *df* = 1, *p* < 0.05). Similarly, when the association between ALR and vocabulary was constrained to be equal between German and Greek, the model fit deteriorated significantly (Δχ^2^ = 4.54, *df* = 1, *p* < 0.05). Taken together, these results indicate that parent teaching was more strongly associated with letter knowledge in Greek than in German, while ALR was more strongly associated with vocabulary in German than in Greek.

Finally, we estimated the indirect effects of HLE on later literacy skills mediated by the emergent literacy skills. The results of mediation analyses for each orthography are shown in Table 5. Parent teaching had an indirect effect on reading via letter knowledge or PA in English and Dutch, while it had an indirect effect on spelling via emergent literacy skills in all languages. Similarly, ALR had an indirect effect on reading via letter knowledge or PA in Dutch and Greek, while it had an indirect effect on spelling via emergent literacy skills in all languages except English. In contrast, shared book reading did not have a significant indirect effect on either literacy outcome.

**TABLE 5:** Indirect effects of HLE on literacy outcomes in each orthography.

	English		Dutch		German		Greek	
	Estimate	95% CI	Estimate	95% CI	Estimate	95% CI	Estimate	95% CI
PT → Voc → Reading	0.00	[−0.02, 0.01]	0.00	[−0.04, 0.02]	0.02	[−0.01, 0.07]	0.00	[−0.01, 0.04]
PT → LK → Reading	**0.03**	**[0.00, 0.08]**	0.05	[−0.01, 0.16]	0.01	[−0.05, 0.08]	0.03	[−0.02, 0.09]
PT → PA → Reading	−0.02	[−0.11, 0.07]	**0.06**	**[0.00, 0.15]**	0.00	[−0.02, 0.04]	0.01	[−0.02, 0.07]
SBR → Voc → Reading	0.00	[−0.01, 0.02]	0.00	[−0.03, 0.04]	0.00	[−0.02, 0.04]	−0.01	[−0.08, 0.01]
SBR → LK → Reading	0.01	[−0.01, 0.04]	0.01	[−0.07, 0.09]	0.00	[−0.07, 0.08]	−0.02	[−0.07, 0.01]
SBR → PA → Reading	0.01	[−0.07, 0.09]	−0.04	[−0.15, 0.01]	0.00	[−0.02, 0.04]	−0.02	[−0.09, 0.03]
ALR → Voc → Reading	0.01	[−0.03, 0.05]	−0.02	[−0.07, 0.02]	0.04	[−0.01, 0.11]	0.01	[−0.01, 0.05]
ALR → LK → Reading	0.02	[−0.01, 0.06]	**0.06**	**[0.00, 0.16]**	0.03	[−0.04, 0.11]	0.02	[−0.01, 0.06]
ALR → PA → Reading	0.05	[−0.03, 0.13]	0.03	[−0.01, 0.12]	0.03	[−0.01, 0.10]	**0.05**	**[0.01, 0.13]**
PT → Voc → Spelling	0.00	[−0.01, 0.03]	0.00	[−0.04, 0.02]	**0.03**	**[0.00, 0.09]**	0.00	[−0.01, 0.04]
PT → LK → Spelling	**0.03**	**[0.00, 0.09]**	**0.05**	**[0.00, 0.13]**	0.01	[−0.08, 0.11]	**0.07**	**[0.02, 0.15]**
PT → PA → Spelling	−0.02	[−0.13, 0.08]	**0.09**	**[0.01, 0.20]**	0.00	[−0.01, 0.03]	0.02	[−0.02, 0.08]
SBR → Voc → Spelling	0.00	[−0.04, 0.01]	0.00	[−0.02, 0.04]	0.00	[−0.03, 0.05]	−0.02	[−0.08, 0.01]
SBR → LK → Spelling	0.01	[−0.02, 0.05]	0.01	[−0.05, 0.08]	0.00	[−0.09, 0.10]	−0.04	[−0.11, 0.00]
SBR → PA → Spelling	0.01	[−0.08, 0.10]	−0.06	[−0.21, 0.00]	0.00	[−0.01, 0.03]	−0.02	[−0.11, 0.04]
ALR → Voc → Spelling	−0.02	[−0.08, 0.01]	−0.02	[−0.09, 0.02]	**0.06**	**[0.01, 0.15]**	0.01	[−0.01, 0.05]
ALR → LK → Spelling	0.02	[−0.01, 0.07]	**0.05**	**[0.00, 0.14]**	0.03	[−0.06, 0.14]	**0.04**	**[0.00, 0.10]**
ALR → PA → Spelling	0.06	[−0.03, 0.15]	0.06	[−0.02, 0.16]	0.01	[−0.02, 0.07]	**0.06**	**[0.02, 0.15]**

*Values shown in bold font are significant at the alpha level of 0.05. CI, confidence interval; PT, parent teaching; SBR, shared book reading; ALR, access to literacy resources; LK, letter knowledge; PA, phonological awareness; Voc, vocabulary.*

## Discussion

The purpose of this study was to examine the longitudinal relations between home literacy environment, emergent literacy skills, and later literacy outcomes across alphabetic orthographies varying in orthographic consistency. By doing so, we aimed to reveal whether and to what extent language and culture (elements of the macrosystem in Bronfenbrenner’s ecological systems theory) can modulate the relation between HLE (microsystem) and literacy development (behavioral outcomes). The results showed first that neither parent teaching nor shared book reading were uniquely associated with emergent literacy skills in English and German, while parent teaching was in Dutch and Greek. It should be noted, however, that the correlation between parent teaching and letter knowledge was significant in English (see Table 4), albeit weak. We should also keep in mind that children’s letter knowledge was assessed with a letter-sound knowledge task instead of letter-name knowledge task in this study. As parents usually teach the names of letters to their child more frequently than teaching the sounds of letters (e.g., Martini and Sénéchal, 2012; Inoue et al., 2018), the observed associations between parent teaching and letter knowledge in this study might be somewhat underestimated. Taken together, our findings suggest that, in line with the predictions of the Home Literacy Model and the findings of previous studies in alphabetic orthographies (e.g., Manolitsis et al., 2009, 2013; Hamilton et al., 2016; Inoue et al., 2018), parent teaching was associated with children’s code-related skills (letter knowledge and PA) in all included orthographies except German. This result differs from previous findings with German-speaking population (Niklas and Schneider, 2013; Niklas et al., 2015). Given our results showing that parents’ teaching was less frequent in German than in all other languages (see Table 1), one possible interpretation would be that German-speaking Austrian parents may follow a low involvement strategy, possibly because they value their child’s autonomy (Ziehm et al., 2013) and rarely think their child needs much help in learning to read and spell before the beginning of Grade 1. Contrary to our expectation, the strongest association between parent teaching and letter knowledge was found in Greek, not in English. This may be at least partly due to the fact that parents’ teaching was the most frequent in English and there was only limited variability on this measure. In fact, the correlations between parent teaching and letter knowledge were of similar magnitude in English, Greek, and Dutch (see Table 4).

Access to literacy resources, on the other hand, was significantly associated with emergent literacy skills in all languages and, as hypothesized, it was uniquely associated with children’s vocabulary knowledge in English, Dutch, and German. The strongest association between access to literacy resources and vocabulary was found in German (see Lehrl et al., 2013, for a similar finding in German). Additionally, access to literacy resources was the greatest and the average score on Vocabulary was the highest in German among the four languages. These results suggest that it may be access to literacy resources rather than parents’ reading to their children that is driving the relation between HLE and vocabulary. In other words, child-initiated activities, in which they can take control of the activity and play, may have a larger impact on their learning than parent-initiated activities (see Grolnick and Ryan, 1987, 1989). This implies that by providing more printed materials at home, parents may increase their children’s opportunities to learn new words and this, in turn, can enhance their child’s autonomy in accessing written materials as well as some active interest in learning new words (for a relevant discussion, see van Bergen et al., 2017). These findings, together with the findings of existing cross-cultural studies (Chiu et al., 2012; Arya et al., 2014; Araújo and Costa, 2015; Lenkeit et al., 2018; Zuilkowski et al., 2019), suggest that there might be value in revising the Home Literacy Model so that ‘presence of reading materials at home,’ which can facilitate child-directed activities, is separated from the ‘shared book reading’ component and becomes part of a broader ‘access to literacy resources’ component.

The results further showed that parent teaching and access to literacy resources had indirect effects on literacy outcomes via emergent literacy skills in all languages, a finding that is consistent with those of previous studies with English-speaking participants (e.g., Hamilton et al., 2016; Inoue et al., 2018). However, it should be noted that the pathways for the indirect effects did not follow the same pattern across languages. More specifically, whereas parent teaching had an indirect effect on reading and/or spelling through code-related skills in English, Dutch, and Greek, its effect on spelling was mediated by vocabulary in German. Similarly, while access to literacy resources had an indirect effect on spelling via vocabulary in German, its effects on the literacy outcomes were mediated by letter knowledge and/or PA in Dutch and Greek. Taken together, our results suggest that the effects of HLE on later literacy development are distributed via more pathways than previously thought, and the possible pathways for the mediated effects are likely to be modulated by language and culture. No particular trend in the role of orthographic consistency in the aforementioned relations emerged, which further suggests that other factors, either distal (e.g., educational context; Arya et al., 2014; Solheim et al., 2020) or proximal (e.g., parenting style; Steinberg et al., 1992; Kiuru et al., 2012) to HLE may account for the observed differences across languages.

An important educational implication of our findings would be to inform parents that increasing access to literacy resources at home may enhance children’s literacy development. However, our results that shared book reading was not uniquely associated with children’s literacy skills suggest that parents may not necessarily know how to effectively engage their children in shared reading activities. Given this, an implication of our findings would be to encourage researchers and educators to suggest the means by which the home literacy activities, shared book reading in particular, could be beneficial for their children’s literacy development (see e.g., Mol et al., 2008; Niklas and Schneider, 2017b; Burgoyne et al., 2018; Noble et al., 2019).

Some limitations of our study are worth mentioning. First, our findings can be generalized only for the age range of the participants in our sample. In order to more fully reveal the relations between HLE and literacy development, future studies should capture longer developmental processes ranging from pre-reading to fluent reading (including measures of reading comprehension) for each language. Second, home literacy activities were assessed retrospectively with a self-report questionnaire to the parents, and this may have resulted in inflated estimates of their literacy-related activities at home due to social-desirability bias (assuming that parents attach a high value to these aspects of home environment). Third, we used observed variables instead of latent variables for HLE and the cognitive constructs in the models, and this might have resulted in the underestimation of the relations between HLE and literacy skills due to measurement error. Fourth, the possible influence of schooling on children’s performance across the testing points was not captured in this study partly because school-level variables were not our primary focus in the present study. Future studies should consider taking school-level variables into account to better understand how schooling, another microsystem affecting children’s literacy development, interact with the effect of HLE. Finally, because developing strictly comparable cognitive and literacy measures across such a diverse group of languages is extremely difficult given the unique features of each language, we decided to use existing measures of cognitive and literacy skills that follow the same administration and scoring procedures across languages. Although the observed differences in the relationships between HLE and literacy outcomes in our study might be partly due to the characteristics of the cognitive and literacy measures used in each language, we also acknowledge that fully controlling for the effect of item characteristics across four diverse languages is almost impossible.

To conclude, the present study examined the developmental relations between HLE, emergent literacy skills, and literacy outcomes in a 2-year longitudinal study with children learning four alphabetic orthographies (English, Dutch, German, and Greek). The results indicated that parent teaching was associated more strongly with letter knowledge and PA in English, Dutch, and Greek, while access to literacy resources is associated more strongly with vocabulary knowledge in English, Dutch, and German. In contrast, the results did not provide evidence for a unique association of shared book reading with cognitive or early literacy skills in any language. Moreover, parent teaching and access to literacy resources had indirect effects on later literacy skills via different emergent literacy skills. These findings suggest that not all HLE components are equally important for specific facets of emergent literacy skills, reading fluency, and spelling across orthographies. The current findings add to the cross-linguistic literature on HLE as this is the first analysis directly comparing associations between HLE and literacy outcomes across different orthographies varying in orthographic consistency.

## Data Availability Statement

The raw data supporting the conclusions of this article will be made available by the authors, without undue reservation, to any qualified researcher.

## Ethics Statement

This study was carried out in accordance with the recommendations of the University of Alberta Human Research Ethics Board with written informed consent from all subjects (parental consent) in accordance with the Declaration of Helsinki. The protocol was approved by the University of Alberta Human Research Ethics Board.

## Author Contributions

TI contributed to the conception and design of the work, ran the analyses, interpreted the findings, and took the lead on writing the manuscript. GM, PJ, and KL organized the data collection at each site, contributed to the interpretation of the findings, and revised the work critically. RP contributed to the conception and design of the work, the interpretation of the findings, and revised the work critically. GG conceptualized the research project, organized the data collection, contributed to the conception and design of the work, and supported the writing of the manuscript. All authors contributed to the article and approved the submitted version.

## Conflict of Interest

The authors declare that the research was conducted in the absence of any commercial or financial relationships that could be construed as a potential conflict of interest.
